# Ovarian Cancer: Latest Advances and Prospects

**DOI:** 10.3390/jcm10245919

**Published:** 2021-12-17

**Authors:** Ludivine Dion, Vincent Lavoué

**Affiliations:** Service de Gynécologie, CHU Rennes, Université de Rennes, F-35000 Rennes, France; ludivine.dion@chu-rennes.fr

The landscape of ovarian cancer therapeutics is experiencing an increase in new opportunities. A great number of new clinical or biological concepts have occurred over recent decades. To address this topic, the present Special Issue in the *Journal of Clinical Medicine (JCM)* is dedicated to showing high-quality scientific publications focusing on surgical insights and medical therapeutics based on new biological classification in managing patients with ovarian cancer.

First, new concepts related to the time of surgery and how to do surgery for women with ovarian cancer are explored in the present special issue. Firstly, for early stage epithelial ovarian cancer (EOC) patients, Merlier et al. [1] compare the survival outcomes of laparoscopic staging with laparotomic staging. Laparoscopy is a good option in the surgical management of early stage EOC, but high-quality staging without compromising safety is required. The role of Minimal Invasive Surgery (MIS) is still a matter of debate in the treatment of early stage EOC patients and no consensus exists concerning the surgical route. In the 2019 ESMO–ESGO guidelines, minimally invasive surgery can be performed for restaging early EOC with a level of evidence IV and a strength of recommendation B. Nevertheless, the standard surgical approach for early EOC is still laparotomy (level of evidence V, strength of recommendation A). In this retrospective study, Merlier et al. [1] report no difference in survival outcomes between laparoscopic and laparotomy approaches. The high-risk of intraoperative tumor rupture is the greatest issue for the use of laparoscopy. However, this risk does also exist with laparotomy. In this study, the laparotomy group showed four tumor ruptures, with none in the laparoscopy group. Among these four ruptures, Merlier et al observed only one death after 92 months of follow-up, with no recurrence or death observed for the three remaining patients (with 31, 44, and 55 month follow-up durations). Thus, laparoscopy and laparotomy display similar oncologic outcomes. MIS may improve patient comfort and delay beginning adjuvant treatment. Thus, laparoscopic staging may be an adequate surgical route for selected patients with early EOC, if performed by well-trained surgeons.

Similarly, in advanced EOC, there are some issues about accurate surgical stadification. There is an absence of a consensus on systematic lymphadenectomy in advanced EOC, since one prospective randomized trial (named the LION study) did not show improvement in overall survival in cytoreductive surgery in the front line. The controversy is due to the fact that that many strategies used neoadjuvant chemotherapy for advanced EOC and reported lymphadenectomy at interval surgical cytoreduction after three chemotherapy cycles. Bund V et al. [2] showed similar results (similar overall survival) when lymphadenectomy was performed during interval debulking surgery after neoadjuvant chemotherapy in patients with initially inoperable advanced EOC, but showed improvement in free disease survival, whatever the number of removed lymph nodes (*p* = 0.005), and whatever their status (metastatic or not). 

Although controversies exist about how to do surgery in early or advanced EOC, this special issue raises the issue of adapting surgery according to frailty in elderly patients. Surgical stress evokes an increased physiological response from frail patients, who are at risk of postoperative complications. Surgeons often choose to simplify surgery in order to decrease the rate of complications, but this choice is often based on chronological age alone without reproducible evaluation of frailty. Dion et al. [3] showed that, among patients with EOC aged ≥70 years, women aged ≥75 years had different treatment to the youngest patient, despite similar tumor characteristics. Of note, the oldest patient received less surgery plus chemotherapy, had fewer bowel resections, received bevacizumab less often and had mono-chemotherapy more often. On analyzing treatments by frailty, surgical complexity was lower in patients with a modified Charlson Comorbidty Index score > 3, showing that surgeons adopt a simpler surgical approach in frailer patients, which could lead to less frequent complications in frailer women (23.5% vs. 35.7%, *p* = 0.223). Thus, chronological age is an insufficient indicator of frailty, but perceived frailty based on subjective criteria may lead surgeons to reduce their “aggressive” surgical approach and potentially reduce the thoroughness of their operative assessment of residual tumor. This “intuitive” approach may also result in the suboptimal application of treatment sequences: primary surgery is too often used over neoadjuvant chemotherapy, which would probably allow frail patients to benefit from combined treatment with chemotherapy and surgery.

Lastly, the future of EOC treatment is probably in the field of biological personalized systemic treatment. Indeed, outcomes could be improved by molecular profiling. Dion et al. [4] conducted a deep review on molecular and genetic alterations in EOC. Carcinogenesis of approximately 15% EOC patients is due to *BRCA1* or *BRCA2* mutation genes. *BRCA* genes are involved in repairing double-strand DNA breaks, making *BRCA*-mutated EOC more sensitive to platinum-based chemotherapy and to PolyAdenosine Diphosphate-Ribose Polymerase (PARP) inhibitors. Olaparib is a PARP inhibitor that showed efficacy in ovarian cancer patients with *BRCA*-mutated genes. PARP inhibitors also demonstrated effectiveness in tumors with a BRCAness (breast cancer) profile. A BRCAness profile is defined by mutations or deficiency in other deficient DNA repair genes. A universally accepted molecular definition of BRCAness is now available to identify optimal theranostic strategies involving PARP inhibitors, such as the HRD (Human Recombination Deficiency) test; however, more specific tests will probably be developed in the future. Besides, TCGA analysis gives rise to the identification of four subgroups of high-grade serous EOC: mesenchymal, proliferative, differentiated, and immunoreactive. These subtypes are not mutually exclusive and are correlated with prognosis. The development of new targeted treatments will probably use this classification, which is not yet used in routine clinical practice. 

Several interesting findings are revealed by this collective work. While biological advances and personalized treatments are addressed in combatting EOC, newer concepts for surgical stadifications (surgical routes, lymphadenectomy, or frailty selection) are also covered. There are still many fundamental questions that require more evidence in order to be answered, related to improving the overall survival of EOC patients.
